# Data on the rootability of *Parkia biglobosa* using pure honey, Coconut Water and Moringa Leaf Extract as an alternative hormones

**DOI:** 10.1016/j.dib.2018.10.002

**Published:** 2018-10-04

**Authors:** O. Dunsin, C.M. Aboyeji, A.O. Adekiya, K.A. Adegbite, O.T.V. Adebiyi, T.O. Adeyemo, A. Joseph, D.M.F. Dunsin

**Affiliations:** a College of Agricultural Sciences, Landmark University, PMB 1001, Omu-Aran, Kwara State, Nigeria; b Landmark University Medical and Health Centre, Landmark University, PMB 1001, Omu-Aran, Kwara State, Nigeria

**Keywords:** Moringa Leaf Extract, Coconut Water, Honey, Rootability, *Parkia biglobosa*

## Abstract

The data article contains the experimental data and figures on the number of rooted cuttings, number of cuttings with callus, cutting mortality and root length of *Parkia biglobosa cuttings.* The investigated data are related to the research article “Effects of alternative hormones on the rootability of *Parkia biglobosa.”* (Dunsin et al., 2014) [1]. In the experimental data, number of rooted cuttings, number of cuttings with callus, number of cuttings with mortality, total number of roots, total root length of cuttings and length of longest root of cuttings data employing alternative hormone (Pure Honey, Coconut Water, Moringa Leaf Extract) on the semi-hardwood stem cutting of *Parkia biglobosa* have been exhibited. The data would be useful to researchers finding alternate growth and rooting hormones that are cost friendly and for vegetative propagation during enrichment planting program of important tree crops that are difficult to propagate via seeds.

## Specifications table

**Table:** 

Subject area	Agronomy
More specific subject area	Physiology/Propagation
Type of data	Tables and Figures
How data was acquired	Propagated in rooting chamber, rooted cuttings, cuttings with callus, cuttings mortality, total roots length and number, were determined
Data format	Raw and analyzed
Experimental factors	Single factor different plant hormone extract (pure honey, coconut water, moringa leaf extract.)
Experimental features	Data collected were subjected to analysis of variance (ANOVA) using SPSS 17.0, and means were separated using Least Significant Difference (LSD).
Data source location	Screenhouse Landmark University Teaching and Research Farm Omu-aran, Kwara- State. Nigeria. Landmark University lies between 8°12׳ N and Longitude 5°08׳ E and is located in the derived savanna ecological zone of Nigeria.
Data accessibility	The data are available within this article
Related research article	Dunsin, O., Ajiboye, G., and Adeyemo, T. (2014). Effect of alternative hormones on the rootability of parkia biglobosa. Journal of Agriculture, Forestry and the Social Sciences (JOAFSS), Vol. 12, No. 2, 2014 [1]

## Value of the data

• Data showed that the rooting ability of *Parkia biglobosa* was successful with the application of alternative hormones that would be an innovative data compared to other researchers.
• The data can be used as a baseline for vegetative propagation of other permanent tree crops that are difficult to propagate via seeds using alternative hormone.
• Investigated data are useful to the researchers working in plant biotechnology, plant physiology, forestry and biochemistry.

## 1 Data

**Fig. 1:**
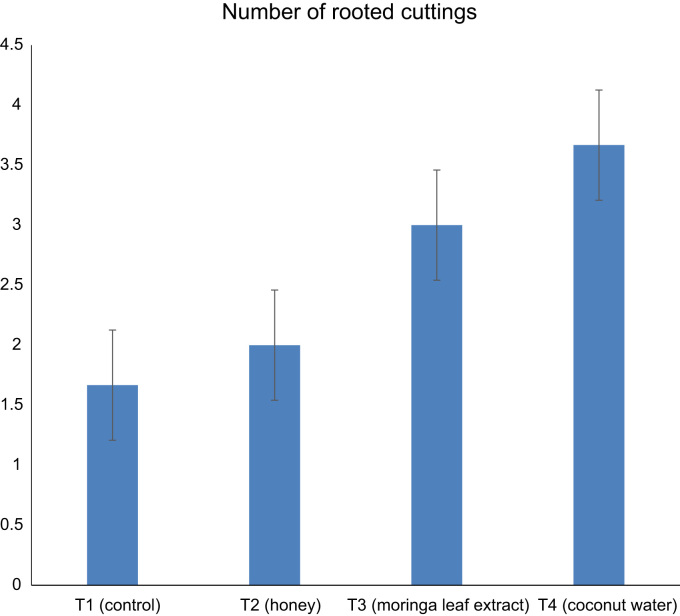
Data showing relationship between the numbers of rooted cuttings among treatments.

**Fig. 2:**
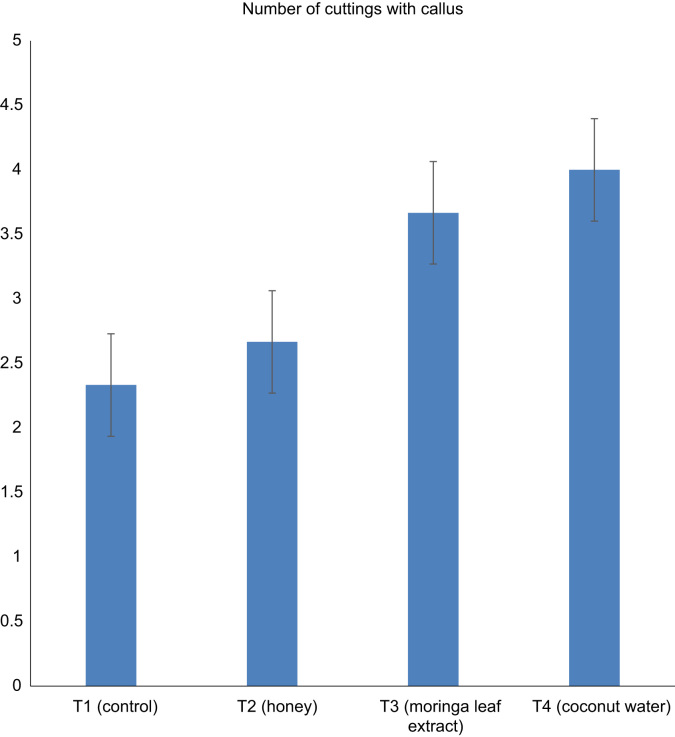
Data showing the number of cuttings with callus among treatments.

**Fig. 3:**
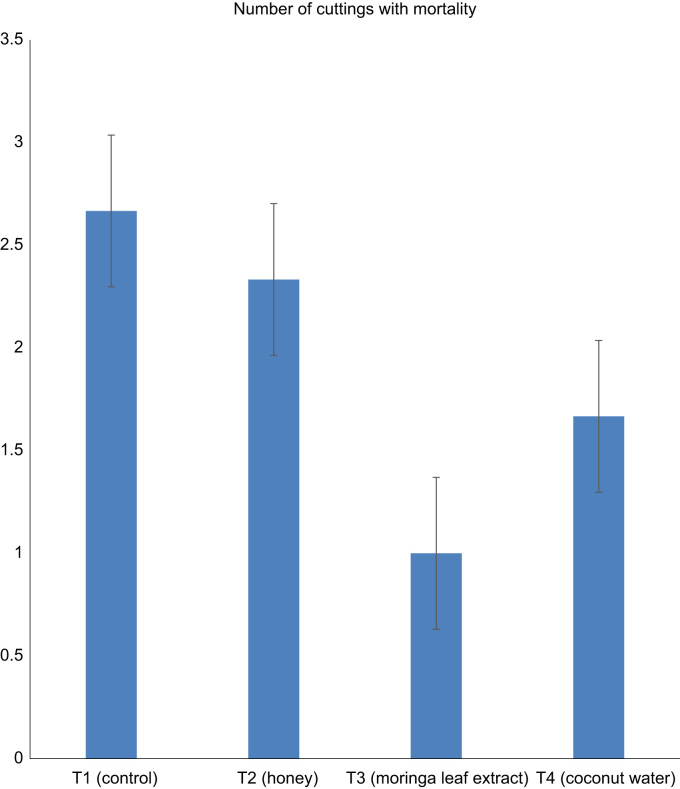
Data showing the number of cuttings with mortality among treatments.

Data in Fig. 1, Fig. 2, Fig. 3 describe the effect of alternative hormones treatment on number of rooted cuttings, callus formation and mortality of *Parkia biglobosa* semi-hardwood cuttings after 28 days of propagation (Fig. 1, Fig. 2, Fig. 3). In Figs. 1 and 2 data, root number and callus formation was influenced by Coconut Water which was supported by the work of Leakey [2]. who noted that, the cuttings that have high level of auxins and cytokinins have higher percentage of rootability. Fig. 3 shows the motility rate of the cuttings. Data in Table 1 explored the number of roots per cutting, total root length and average root length of cuttings from semi-hard wood of *parkia biglobosa*.

**Table 1:** Effect of alternative hormones on total number of roots, total root length, and length of longest root per cutting of *Parkia biglobosa* semi-hardwood cuttings (day 28).

Treatments	Total number of roots	Total root length	Length of longest root
*T*1 (control)	3.00	4.047	1.407
*T*2 (honey)	5.333	7.470	1.427
*T*3 (moringa leaf extract)	8.667	12.457	1.493
*T*4 (coconut water)	7.333	11.617	1.487
MEAN	6.083	8.898	1.454
%CV	40.605	43.83	2.958
LSD	3.245	5.342	0.083

## 2 Experimental design, materials and methods

### 2.1 Site description and treatments

An experiment was carried out at Landmark University Teaching and Research Farm Omu-aran, Kwara inside a three polypropagator rooting chambers fabricated following the design described by Leakey et al. [2] from February 2014 to April 2015. The study area is located at latitude 8°12׳ N and Longitude 5°08׳ E, with altitude of 506 m above sea level of the Guinea savannah zone of Nigeria. Vigorous and disease free cuttings were taken from the mother tree of *Parkia biglobosa* in Landmark University Farm area. All cuttings materials were taken from a single mother tree early in the morning, with the aid of a sterile secateurs, cuttings were separated to prevent the xylem from being crushed, given it a very smooth surface and prevent microbial infection. The stem cuttings obtained were semi-hardwood cutting of uniform diameter and length of 15 cm in an angle of 45° with at least 2 buds on each stem and all leaves were removed to reduce evaporation from the cuttings. Cuttings were treated and soaked for 15 min in a solution of fungicide fungu force. Three types of alternative rooting hormones namely; Honey (Pure Honey), Coconut Water and Moringa Leaf Extract (MLE) were used. The stem cuttings were treated with the alternative hormones for 3 min and air dried for 5 min after each basal end treatments. The design of the experiment was a Randomized Complete Block Design (RCBD) where five cuttings of *Parkia biglobosa* were subjected to four treatments (control included) and replicated three times to give a total of 60 cuttings. At the end of the experiment, the following observations were made and data collected were; Number of rooted cuttings, Number of cuttings with callus, Number of cuttings with mortality, Total number of roots, Total root length of cuttings and Length of longest root of cuttings.

### 2.2 Statistical analysis

The data collected was analyzed using Analysis of Variance (ANOVA) and the means were separated using Least Significant Difference (LSD).

## References

[1] Dunsin O., Ajiboye G., Adeyemo T. (2014). Effect of alternative hormones on the rootability of parkia biglobosa. J. Agric. For. Social Sci. (JOAFSS).

[2] Leakey R.R.B., Mesén J.F., Tchoundjeu Z., Longman K.A., Dick J.McP., Newton A.C., Matin A., Grace J., Munro R.C., Muthoka P.N., Rogers M.T. (1990). Low-technology techniques for the vegetative propagation of tropical trees.

