# Supplementary material for: Data on the rootability of Parkia biglobosa using pure honey, Coconut Water and Moringa Leaf Extract as an alternative hormones
Source: Data Brief. 2018 Oct 4;21:511–5. doi: 10.1016/j.dib.2018.10.002 (PMC6199776; doi:10.1016/j.dib.2018.10.002)
Supplement: Supplementary file 1 — Supplementary material [file mmc1.docx]

None declared.
